# Lipid monitoring using non-invasive measurement technologies and machine learning: a systematic review

**DOI:** 10.1007/s00404-025-08254-6

**Published:** 2026-01-30

**Authors:** Julia Endrass, Valerija Krbanjevic, Kerstin Khattab, Elena Pavicic, Michelle Zwahlen, Petra Stute

**Affiliations:** 1 Faculty of Medicine, University of Bern, Bern, Switzerland; 2 Department of Cardiology, Cardiance Clinic, Pfaeffikon, Switzerland; 3 Department of Obstetrics and Gynaecology, University Hospital Bern – Inselspital, Bern, Switzerland; 4 Graduate School for Health Sciences, University of Bern, Bern, Switzerland; 5 Department of General Internal Medicine, Hospital Thun, Thun, Switzerland

**Keywords:** Wearable devices, Lipids, Cardiovascular risk, Machine learning in healthcare, Preventive cardiology, Non-invasive monitoring

## Abstract

**Background:**

Cardiovascular diseases (CVD) are the leading cause of death among women, with risk increasing after menopause. Lipid levels are key biomarkers, yet conventional blood tests remain invasive and underutilized. Non-invasive technologies and machine learning (ML) may offer new approaches to lipid monitoring and risk assessment using wearable devices and biosensors.

**Objective:**

This systematic review investigates the availability, accuracy, and clinical applicability of minimally and non-invasive lipid monitoring methods and ML-based cardiovascular risk estimation in adults.

**Methods:**

A systematic search was conducted in MEDLINE, Embase, Cochrane Library, Web of Science, Scopus, and ClinicalTrials.gov (2010–2024). Studies in English were included; case reports and animal studies were excluded. Data extraction focused on devices, measurement approach, and predictive utility for cardiovascular outcomes. Methodological heterogeneity was addressed through narrative synthesis and thematic grouping (Thomas in Cochrane Handb Syst Rev Interv, 2024).

**Results:**

From 14,863 records, 37 studies were included. Near-infrared, saliva-based, and smartphone-enabled fingertip devices showed promising accuracy. ML models using wearable-derived physiological data demonstrated moderate success in predicting cardiovascular risk and lipid levels.

**Conclusion:**

Minimally and non-invasive lipid monitoring and ML-based risk prediction may support accessible, personalized cardiovascular risk management. Despite encouraging findings, validation in large-scale, long-term studies is essential before clinical adoption.

**Trial registration:**

Title registration number (on PROSPERO): CRD420251105896

**Supplementary Information:**

The online version contains supplementary material available at 10.1007/s00404-025-08254-6.

## Introduction

High blood cholesterol, including high levels of Low-Density Lipoprotein Cholesterol (LDL-C), contributes to the increase of CVDs and is referred to as hypercholesterolaemia. CVDs remain the leading cause of death worldwide. In 2022, an estimated 19.8 million people died from CVDs, representing 32% of all global deaths, and 85% of these were due to heart attack and stroke [2]. In women, CVD becomes the leading cause of death after menopause, highlighting the importance of early detection and prevention [3]. Effective control and detection of pathogenic factors in an early stage may delay or even prevent the development of atherosclerosis and the onset of cardiovascular disease [4].

Established models for cardiovascular risk assessment face several challenges. For instance, tools like the ESC SCORE2, commonly used in Europe, require blood markers like Total Cholesterol (TC) and High-Density Lipoprotein Cholesterol (HDL-C), as well as other clinical evaluations [5]. Standard methods of monitoring blood cholesterol levels require invasive techniques by taking blood samples, which can be painful and uncomfortable [6, 7]. These risk models frequently overlook important lifestyle factors, such as stress and sleep quality, as well as gender-specific risk aspects, like hormonal changes during menopause [8, 9].

The drop of estrogen after the menopausal transition is associated with reduced vascular elasticity, endothelial dysfunction [10], oxidative stress [11], and adverse changes in the lipid profile. The pathophysiological processes promote chronic inflammation, atherogenesis [12], and unfavorable metabolic alterations, such as increased TC, triglycerides (TG), visceral adiposity, and a higher risk of insulin resistance along with type 2 diabetes [13].

This systematic review examines the current landscape and clinical applicability of minimally and non-invasive technologies for lipid profiling and cardiovascular risk evaluation in adults. The challenges associated with cardiovascular health highlight the need for minimally and non-invasive, accessible, and continuous methods of risk assessment. Such approaches may enhance early detection, prevention, and contribute to more effective and individualized healthcare [11].

## Methods

### Study protocol

The protocol for this systematic review was registered in PROSPERO (registration number: CRD420251105896) in accordance with PRISMA 2020 guidelines [14]. The full protocol is publicly available in the PROSPERO database: https://www.crd.york.ac.uk/PROSPERO/view/CRD420251105896. The original internal protocol included only the initial search in 2022. Prior to finalizing the review, an updated search was conducted in 2024 and documented in the PROSPERO registration to ensure inclusion of the most recent evidence. No other changes were made to the review protocol.

### Search strategy

The methodology followed Cochrane guidance and PRISMA 2020 reporting standards. A comprehensive search was conducted in MEDLINE All (Ovid), Embase (Ovid), Cochrane Library (Wiley), Web of Science Core Collection (Clarivate), such as Science Citation Index Expanded (SCIE) & Emerging Sources Citation Index (ESCI), Scopus (Elsevier), and ClinicalTrials.gov (National Library of Medicine). The search strategy was based on controlled vocabulary (e.g., MeSH, Emtree) and free text terms, covering cardiovascular disease, risk assessment, lipids, and wearable technology. The first search was conducted in September 2022 and updated in May 2024.

The initial search strategy was designed to investigate wearable technologies for the assessment of both blood pressure and lipid status in the context of cardiovascular risk prediction tools. Due to the breadth of the topic, this review was subsequently divided into two separate publications—one focused on blood pressure assessment and the other on lipid status. This decision aligns with Cochrane guidance, which recommends splitting a review when the scope becomes unmanageable or when distinct sets of interventions, populations or outcomes are best addressed separately [1]. This manuscript reports exclusively on the subset of studies related to lipid monitoring. Accordingly, all results, analyses, and risk-of-bias assessments presented in this paper refer solely to the lipid-related studies.

### Definition of terminology and machine learning concepts

For clarity and consistency, key terms are defined below. Non-invasive methods do not require penetration of the skin or extraction of bodily fluids. Minimally invasive methods involve minor disruption of tissues, such as finger-prick blood sampling or saliva collection.

To support readers less familiar with data science, commonly referenced ML concepts are briefly summarized. Random forests and other ensemble methods aggregate weaker models (e.g., decision trees) to improve performance and reduce overfitting. Support vector machines (SVMs) classify observations by identifying a decision boundary that maximizes the margin between classes. K-nearest neighbors (KNN) assigns labels based on the majority class among nearby data points. Extreme gradient-boosting (XGBoost) is a high-performance gradient-boosting framework widely used for structured clinical datasets. Long short-term memory networks (LSTMs) are recurrent neural networks designed for sequential data such as wearable signals. Generalized linear models (GLMs), including logistic regression, often serve as baseline comparators in ML studies. Stacking models combine several base learners using a meta-model to improve overall prediction.

For time-series forecasting, auto-regressive integrated moving average (ARIMA) models are sometimes compared with more complex neural architectures. The Framingham Risk Score (FRS) represents a traditional 10-year CVD risk calculator based on age, sex, blood pressure, cholesterol levels, smoking, and diabetes. The area under the receiver operating characteristic curve (AUC) reflects a model’s ability to discriminate between outcome classes, with 0.5 indicating no discrimination, values from 0.7–0.8 acceptable, > 0.8 good and > 0.9 excellent. Mean absolute error (MAE) and root mean square error (RMSE) quantify prediction error, with RMSE penalizing larger deviations more strongly than MAE. Sensitivity and specificity describe the proportion of correctly identified positive and negative cases, respectively. Accuracy reflects the overall proportion of correct classifications but may be misleading in imbalanced datasets. The Brier score assesses the accuracy and calibration of predicted probabilities, with lower values indicating better performance.

### Inclusion and exclusion criteria

Inclusion criteria:

- Studies assessing wearable devices for blood lipid measurement.
- Studies evaluating the predictive value of wearable data for cardiovascular risk and lipid profile assessment.
- Original research, preprints, and systematic reviews published in English from 2010 to 1 May 2024.

Exclusion criteria:

- Animal studies.
- Studies in languages other than English, due to limited translation feasibility and to ensure consistent methodological assessment.
- Case studies and non-peer-reviewed articles.

### Screening and data extraction

The title and abstract screening were conducted in Covidence [15] by independent reviewers, following a dual control principle. Full-text screening was performed based on the mentioned eligibility criteria. Data extraction was done by two independent reviewers. Device type, measurement method, validation against traditional lipid measures, and predictive utility for cardiovascular risk assessment were noted in an Excel sheet. As correlation coefficients and mean absolute error (MAE) were stated in most papers, these values were used for comparability. Given the broad scope of the review question, considerable clinical and methodological heterogeneity was observed across studies. While this limited the feasibility of a quantitative synthesis, it was addressed through structured narrative synthesis and careful subgroup consideration, in line with guidance from the Cochrane Handbook (Sect. 2.3.2) [1].

### Quality assessment

Study quality was assessed using appropriate risk-of-bias tools, including the Cochrane Risk of Bias tool for randomized trials [16] and the Newcastle–Ottawa Scale [17] for observational studies.

### Search date and timeline

The last literature search was conducted on 1 May 2024. Given the high number of studies retrieved (14,863 records after removal of duplicates), the screening and data extraction stages were particularly time-consuming and required significant collaborative effort.

The resulting time gap between the search date and manuscript submission is therefore acknowledged and reflects the methodological thoroughness of the review. All steps were conducted and reported in line with Cochrane and PRISMA 2020 standards [18].

## Results

This review is part of a broader research project on cardiovascular biomarkers, with a companion paper focusing on blood pressure measurement via wearables. The initial literature search was performed in September 2022 and was updated on 1 May 2024. Of 22,903 records identified, 14,863 remained after deduplication. Screening was conducted jointly for the overall project, resulting in 289 full-text studies. Of these, 37 met the predefined criteria specific to lipid monitoring and constitute the dataset for the present review. All analyses, narrative synthesis, and risk-of-bias assessments presented in this manuscript therefore refer exclusively to these 37 lipid-related studies.

A summary of key device types, sample sizes, and headline performance metrics is provided in Table 1 to complement the detailed extraction presented in Supplementary File 4.

**Table 1:** Summary of device types, study samples, and headline performance metrics among included lipid-related studies

Technology category	Study	Sample size	Measurement approach	Key performance metrics for lipids
Non-invasive NIR^1^ optical cholesterol sensor	Ahniar et al. 2022	*N* = 5	Prototype photodiode-based NIR sensor (940 nm) measuring cholesterol non-invasively through tissue and compared with invasive standard	Average error 1.46%; accuracy 98.54%; R^2^ = 0.9878 vs gold standard invasive cholesterol measurement
Non-invasive IoT^2^ multisensor device	Darmawan et al. 2022	*N* = not reported	IoT home-check-up device using MAX30105 sensor for non-invasive cholesterol, glucose and uric acid from finger, data sent to database/app	Cholesterol accuracy 91% vs reference device, requires further calibration for clinical use
Saliva electrochemical biosensor	Eom et al. 2020	*N* = 3	Disposable platinum nanocluster enzyme-based EC^3^ sensor measuring cholesterol in saliva	Linear range 2–486 µM; limit of detection ≈2 µM; sensitivity 132 µA·mM⁻^1^·cm⁻^2^; demonstrating feasibility, broader clinical testing recommended
Saliva microfluidic sensor	Vinoth et al. 2023	*N* = not reported	3D-printed microfluidic device with integrated porous membrane and enzyme-based EC sensors for multiplex salivary biomarkers (incl. cholesterol)	Saliva cholesterol detected at < 15 µM; repeatable and stable; good agreement with reference methods and correlated with HPLC^4^; overall device showed reliable multiparametric performance
Smartphone-integrated EC TG^5^ analyzer	Wang et al. 2019	*N* = 106	Smartphone-integrated EC analyzer with disposable TG/TC^6^ test strips using 1–2 µL finger-prick blood	For TG: highly linear vs clinical analyzer (R^2^ = 0.989); reproducibility: CV ~ 2–4%; minimum detectable TG difference 0.01 mmol/L; similar approach used for TC monitoring
Smartphone-based colorimetric analyzer	Oncescu et al. 2014	*N* = 9	Smartphone accessory (smartCARD) imaging standard colorimetric cholesterol test strips and analyzing color via app	Deviation ≤ 5.5% vs CardioChek PA; inter-phone variation < 5.8%; typical per-test accuracy < 1.8% for TC quantification
Smartwatch-based CVD^7^ prediction using ML^8^	Kim et al. 2021	*N* = 6,170	ML model using variables compatible with Samsung Smartwatch (BP, pulse, glucose, BMI, stress etc.) to predict several CVD-related conditions including dyslipidemia	The Gradient Boosting Machine performed best: accuracy 0.874, AUC^9^ 0.850, sensitivity 0.788, specificity 0.882 for composite CVD conditions; no separate dyslipidemia-specific metrics reported
Smartwatch-based MedAI framework	Himi et al. 2023	*N* = 278	Smartwatch with 11 sensors and ML algorithms (RF^10^, SVM^11^, KNN^12^, XGBoost^13^, LSTM^14^) to predict 12 diseases including dyslipidemia	RF reached overall accuracy 99.4% across all diseases; no separate performance metrics reported specifically for lipid disorders
Automated ML (AutoML) for CVD risk	Alaa et al. 2019	*N* = 423,604	AutoPrognosis automated ML pipeline using 473 variables, including cholesterol, for 5-year CVD risk prediction	ML-based model achieved AUC 0.774 vs 0.724 for Framingham; no separate lipid-specific performance metrics were reported
AI^15^ for coronary artery disease prevention	Chen et al. 2023	Review	Summarizes recent advances in AI for risk assessment of primary prevention of coronary artery disease	AI offers a multimodal risk prediction, gaps remain in clinical validation, not specific for lipids
Wearable-based ML ensemble model	Huang et al. 2022	*N* = 600	Ensemble ML models combining lifestyle questionnaires, clinical blood tests (including lipids), ambulatory BP^16^/HR^17^ and Fitbit activity data for CVD risk	MLAs^18^ outperformed Framingham risk score (AUC up to ~ 0.79 in combined models). Lipid variables were part of the predictors, but no separate performance metrics were reported
Wearable-based ML stacking model	Liu et al. 2022	*N* = 918	Stacking ensemble of ML algorithms on public CVD datasets that include BP and cholesterol among input features	Stacking model achieved AUC 0.927 and accuracy 88.1% for CVD classification; cholesterol was included as a feature, but no lipid-specific prediction metrics were reported
Smartphone-based ML risk model including TC	Seto et al. 2020	*N* = 5,992	ML models (e.g., RF, GLM^19^, boosting) using demographic, diet, physical activity and mental health variables to predict CVD risk factors including TC	Highest accuracy 73% for hypertension prediction using RF; TC was one of the risk factors modeled, no explicit performance metrics reported for lipid prediction
EHR^20^-based ML for familial hypercholesterolaemia	Akyea et al. 2020	*N* = 4,027,775	Five ML algorithms applied to primary care records (incl. TC) to detect familial hypercholesterolaemia	All ML models except logistic regression achieved AUC > 0.89 for FH^21^ detection (logistic regression AUC 0.81); ensemble model had positive likelihood ratio 45.5, indicating strong performance for lipid disorder case-finding
NIR + time-series models for cholesterol prediction	Krishnamoorthy et al. 2024	*N* = 1	Prototype NIR device for non-invasive glucose and cholesterol monitoring; time-series prediction using ARIMA^22^ vs LSTM	ARIMA showed substantially lower RMSE^23^ than LSTM (≈50.3% lower for cholesterol)
Non-invasive radial pulse-wave analysis	Qi et al. 2020	*N* = 523	Radial pulse-wave signals plus brachial–ankle PWV^24^, analyzed against metabolic risk factors including TC, LDL-C^25^, HDL-C^26^ and TG	Model including pulse-wave feature h3/h1^27^ improved AUC (0.86 vs 0.84); NRI 50%; IDI^28^ 3.16%; decreases in specific waveform parameters associated with higher TC, LDL-C, UA^29^ and lower HDL-C, supporting links between vascular signals and lipid status
ML-based CVD prediction using wearable data	Zhou et al. 2022	*N* = 692	Extraction of 66 high-resolution digital phenotypes from Fitbit Charge HR; ML models relating features to cardiometabolic risk markers including lipid abnormalities	High-resolution HR features improved prediction of lipid abnormalities and obesity, with Brier score improvements of ~ 17.9–22% compared with baseline models using age, sex and resting HR
Wearable data and other clinical and lifestyle data for cardiovascular/metabolic risk prediction	Lim et al. 2018	*N* = 233	Fitbit Charge HR-derived HR and step counts combined with clinical lipids, glucose and lipidomics for association analysis	Resting HR positively associated with TG, FPG, TC and blood pressure; activity (steps) associated with lipidomic sphingolipid profiles and metabolic markers; no explicit lipid prediction model developed
Wearable HR + step-based deep learning	Ballinger et al. 2018	*N* = 14,011	Semi-supervised LSTM model (“DeepHeart”) using HR and step data from consumer wearables plus self-reported diagnoses	For high cholesterol classification: AUC 0.7441; deep learning outperformed traditional ML baselines (e.g., logistic regression) despite weak direct correlation between HR variability and cholesterol
Smart Wearable with ML	Damre et al. 2022	*N* = not reported	Wearable device measuring HR, BP, SpO₂ and temperature; ML algorithms detect early deviations from individual baselines and generate health alerts	Demonstrated conceptual feasibility of ML-based early detection across multiple conditions; no quantified lipid-specific performance metrics reported

^1^NIR = near-infrared

^2^IoT = internet of things

^3^EC = electrochemical

^4^HPLC = high-performance liquid chromatography

^5^TG = triglycerides

^6^TC = total cholesterol

^7^CVD = cardiovascular disease

^8^ML = machine learning

^9^AUC = area under curve

^10^RF = random forest

^11^SVM = support vector machine

^12^KNN = k-nearest neighbors

^13^XGBoost = extreme gradient-boosting

^14^LSTM = long short-term memory network

^15^AI = artificial intelligence

^16^BP = blood pressure

^17^HR = heart rate

^18^MLA = machine learning algorithms

^19^GLM = generalized linear model

^20^EHR = electronic health record

^21^FH = familial hypercholesterolaemia

^22^ARIMA = auto-regressive integrated moving average

^23^RMSE = root mean square error

^24^PWV = pulse-wave velocity

^25^LDL-C = low-density lipoprotein cholesterol

^26^HDL-C = high-density lipoprotein cholesterol

^27^H3/h1 = ratio of the third to the first harmonic amplitude in the radial pulse waveform, a feature commonly used to characterize vascular stiffness and arterial waveform morphology

^28^IDI = integrated discrimination improvement

^29^UA = uric acid

### Risk-of-bias*** assessment***

Risk-of-bias assessment demonstrated substantial methodological heterogeneity across the 37 studies. Prototype studies using infrared sensors, saliva biosensors, or smartphone-based electrochemical devices typically had high or unclear risk of bias due to very small sample sizes, convenience sampling, limited reporting of participant selection and lack of blinding or standardized outcome assessment. Observational and ML studies showed moderate risk, with common issues including volunteer-based cohorts, self-reported diagnoses, insufficient confounding control and limited external validation. Only a few large, registry-based studies had lower risk, although generalizability. These concerns were considered when interpreting the findings, with greater weight placed on more rigorous study designs. A detailed overview of individual risk-of-bias ratings is provided in Supplementary Table 4.

### Innovative methods for cholesterol measurement

Cholesterol measurement methods can be categorized into invasive, minimally and non-invasive approaches [19]. The current gold standard remains invasive blood sampling with laboratory-based enzymatic assays TC, LDL-C, HDL-C and TG [7]. Minimally invasive methods use alternative bodily fluids like saliva or tears to detect lipid-related biomarkers through biochemical or enzymatic processes [19]. Non-invasive technologies aim to estimate lipid profiles from external physiological measurements, offering a potentially safer, more convenient option for continuous monitoring [20]. The following section outlines innovative approaches to minimally and non-invasive cholesterol assessment.

### Infrared/skin sensors

Several non-invasive methods for cholesterol detection use infrared (IR) and near-infrared (NIR) spectroscopy, with effective wavelengths between 700 and 1400 nm. In particular, 850 nm, 940 nm, and 1200 nm have been identified as most effective for assessing cholesterol levels [6, 20].

Ahniar et al. (2022) proposed an NIR sensor for non-invasive cholesterol measurement, developing a hardware prototype that detects blood flow changes through skin tissue and achieved 98.54% accuracy in 20 participants. However, performance was affected by finger thickness and component tolerances [20].

Darmawan et al. (2022) developed a non-invasive Internet of Things (IoT)-based device capable of measuring body temperature, uric acid, cholesterol and blood glucose levels at home. Users enter basic demographic data and physiological conditions (e.g., fasting state) before measurement. The system achieved a cholesterol measurement accuracy of 91%, but sensor signal interference and incomplete specification of the optimal IR-wavelengths for simultaneous measurement of multiple biomarkers limit its current clinical applicability [19].

### Saliva sensors

Saliva has emerged as a promising bodily fluid for minimally invasive cholesterol detection, as collection is simple and painless compared with blood sampling [21]. However, its lower cholesterol concentrations (10–320 µM vs. < 5.17 mM in serum), pose challenges for accurate measurement [21]. Eom et al. (2020) developed an enzyme-based electrochemical biosensor using platinum nanoclusters (Pt-NCs), capable of detecting concentrations as low as 2 µM in hyperlipidemic patients, though further validation is required [21].

Similarly, Vinoth et al. (2023) introduced a portable microfluidic sensor enabling wireless transmission and simultaneous biomarker detection in saliva, including cholesterol. These innovations highlight the growing potential of saliva-based, point-of-care cholesterol monitoring systems, while clinical applicability remains to be established [22].

### Smartphone-integrated devices

Smartphone-integrated devices allow cholesterol and TG measurement from fingertip capillary blood and may be useful for patients requiring frequent monitoring when venous sampling is impractical [23].

Wang et al. (2019) developed a miniaturized electrochemical analyzer integrated into a smartphone, processing signals from disposable test strips and displaying results with the option to share them with healthcare providers. Fu et al. (2018) presented a similar system for TC detection, with one finger pricked whole blood drop. Both authors showed good agreement with standard laboratory analyzers in healthy and hyperlipidemic participants [23].

Oncescu et al. (2014) proposed a smartphone-based colorimetric, where enzymatic reactions produced visible color changes on test strips. A calibrated reaction time improved accuracy, reducing deviations to 1.8% across physiological range (140–400 mg/dl). These findings suggest that smartphone-integrated devices, though not non-invasive, may expand access to lipid testing and support patient self-monitoring [24].

### Machine learning

The healthcare sector is undergoing a transformation, increasingly shaped by concepts, such as the digital health and the IoT with advances in wearable technologies, enabling continuous monitoring of heart rate variability, physical activity, sleep, oxygen saturation and other physiological signals [24–28]. Parallel progress in ML allows complex patterns to be extracted from heterogeneous longitudinal datasets, supporting personalized risk prediction and early disease detection [29–31]. Combined, wearable sensing and ML offer opportunities for individualized and proactive health management [32, 33].

Wearables have demonstrated reliability for heart rate and arrhythmia monitoring, and can support metabolic disease management, by generating objective, longitudinal health measures that complement subjective reports when integrated with ML techniques [27, 28, 34].

### Machine learning for cardiovascular risk prediction

Within cardiovascular research, ML has been widely applied to improve risk prediction, facilitate early detection and personalize preventive strategies. Techniques, such as random forests, support vector machines, deep neural networks, and ensemble learning models, have outperformed traditional risk scores [35–37].

Kim et al. (2021) developed a CVD prediction model using data from the Korean National Health and Nutrition Examination Survey, adapted for smartwatch users. The authors showed that wearable-compatible indicators combined with ML achieved strong predictive performance [38]. Similarly, new mobile health applications including “MedAI” developed by Himi et al. (2023), exemplify the expansion of smartphone- and wearable-based prediction models [39]. Early frameworks such as “AutoPrognosis” by Alaa et al. (2018) used electronic health records without real-time physiological signals [40]. Similarly, Chen et al. (2023) highlighted that most AI-driven models for coronary artery disease prevention rely on conventional clinical variables [37].

More recent studies have begun integrating wearable-derived features: Huang et al. (2022), used heart rate variability and physical activity to enhance ensemble ML models [41], and Liu et al. (2022) improved prediction via stacking techniques [36].

Despite progress, estimating metabolic biomarkers such as lipid profiles from wearable data remains at an early stage. Seto et al. (2020) applied demographic and lifestyle variables to predict cardiovascular risk factors, with limited success for lipids [42]. Akyea et al. (2020) employed supervised ML models to enhance the detection of familial hypercholesterolaemia in primary care, achieving superior performance compared to traditional diagnostic criteria. However, their approach still depended on laboratory-measured lipid values, illustrating that fully non-invasive prediction remains elusive [43]. First exploratory studies, e.g., Krishnamoorthy et al. (2024), attempted to predict lipid parameters directly from wearable data, though accuracy remained insufficient [44].

### Key studies on wearable-based lipid risk prediction

Qi et al. (2020) explored whether specific radial pulse-wave variables, in combination with brachial-ankle pulse-wave velocity (Ba-PWV), could serve as indicators for metabolic risk factors, including lipid levels, and improve the prediction of hypertension. Their study analyzed a cohort of 523 participants with complete non-invasive measurements of radial pulse wave and Ba-PWV, as well as blood biomarkers. Several pulse-wave parameters showed significant independent associations with lipid profiles, suggesting that different components of the pulse waveform may reflect distinct metabolic processes. The authors propose that these wearable-compatible signals may capture macrovascular and microvascular changes associated with metabolic syndrome, which may in turn affect lipid homeostasis. Although the study did not include machine learning or attempt to predict exact lipid concentrations, it provides compelling evidence that vascular signals captured non-invasively may serve as supplementary indicators for metabolic health screening [45].

Zhou et al. (2022) investigated whether high-resolution heart rate data from consumer wearables could predict cardiometabolic risk markers, with a focus on lipid profiles. Participants wore devices that continuously recorded heart rate, while laboratory assessments provided lipid panels. Features from the recordings were analyzed with random forest models stratified by activity state. Sedentary-state data yielded the highest predictive accuracy, suggesting that resting physiological signals may better capture lipid-related autonomic patterns than those recorded during activity. Although accuracy did not reach clinical thresholds, this study provides early evidence that passive heart rate monitoring combined with ML could contribute to non-invasive lipid risk assessment, pending further model refinement and validation [46].

Lim et al. (2018) investigated wearables in cardiovascular and lipidomic research using 233 healthy participants in the “SingHEART/BioBank” study. Over four days, Fitbit Charge HR devices recorded step counts and heart rate, complemented by clinical assessments including lipid profiles, FPG, blood pressure, anthropometrics, lifestyle questionnaires, cardiac imaging, and lipidomic analysis. Resting heart rate (RHR) was positively associated with BMI, waist circumference, blood pressure, TG and FPG, while associations with LDL-C, HDL-C or TC were inconsistent. Daily steps showed limited associations, mainly linking higher activity with lower BMI, waist circumference, and TG in male participants. A subset analysis found step counts correlated with reduced sphingolipid, whereas resting heart rate did not. Although the study did not attempt lipid prediction, it demonstrated that consumer-grade wearables can provide valuable insights into both physiological and lipid-related aspects of cardiometabolic health [47].

Ballinger et al. (2018) investigated the potential of using wearable data to estimate cholesterol levels through ML. Using “DeepHeart”, a semi-supervised deep learning model trained on data from over 14,000 participants (heart rate, step count and activity levels) the study combined physiological signals with clinical information to predict medical diagnoses. Even without pretraining, the model outperformed traditional baselines such as logistic regression, achieving an area under the curve (AUC) of 0.67 for high cholesterol classification. The most accurate results for cholesterol detection were obtained through a semi-supervised training strategy, reaching an AUC of 0.74. Interestingly, cholesterol showed the weakest direct link to heart rate variability compared with the other health conditions, suggesting performance relied partly on indirect indicators, such as age, sex, or medication. This model also confidently classified many participants as low risk, indicating potential to reduce unnecessary follow-up testing. While refinement is still needed, this study provides early evidence that wearable-derived signals, combined with advanced ML, may support non-invasive cardiovascular and metabolic risk screening [48].

## Discussion

Emerging technologies are reshaping non-invasive cardiovascular risk assessment and lipid monitoring. This review examines the possibility and availability of estimating lipid profiles using minimally and non-invasive techniques, as well wearable-derived physiological data interpreted using ML algorithms.

Non-invasive cholesterol sensors based on IR and NIR spectroscopy may improve user convenience and monitoring frequency, yet challenges, such as sensor calibration, signal interference, and tissue variability, still limit clinical applicability [18, 19, 49]. Minimally invasive saliva-based approaches offer a patient-friendly alternative, but evidence remains insufficient, and correlations between salivary and serum lipid levels need further validation through larger, standardized clinical studies [21, 22].

Parallel to these advancements, digital health technologies including smart wearable devices, ML, and AI are rapidly transforming cardiovascular risk assessment. Approaches like transfer learning (TL), as discussed by Chato et al., offer strategies for improving model performance in data-limited settings, while smart wearable technologies enable continuous, non-invasive monitoring of physiological parameters and direct integration of ML directly into everyday health tracking [49].

An illustrative example of this convergence is the smart wearable developed by Damre et al. (2022), which combines wearable technology with adaptive ML algorithms for early disease detection. The device continuously monitors vital signs and identifies deviations from individual baseline patterns, providing health recommendations that could facilitate earlier intervention and potentially improve treatment outcomes and reducing healthcare costs [50]. Despite substantial technological progress, the clinical translation of non-invasive lipid monitoring and ML-based lipid prediction is still limited [45, 46]. Most approaches remain in preclinical or early validation stages, with few achieving the level of clinical testing necessary for regulatory approval. Additionally, the application of wearable-derived data for lipid prediction is still largely unexplored, and existing ML models often lack the robustness and reproducibility needed for clinical use [42–44].

Among the studies discussed, correlations have been identified between patients’ sedentary [46] and resting [47] heart rate signals, vascular measures, such as diastolic duration [45], brachial-ankle pulse-wave velocity [45], and their lipid profiles, particularly TC, LDL-C, and HDL-C. Ballinger et al. reported that among the various biomarkers measured, cholesterol levels showed the weakest correlation with heart rate variability which makes their findings particularly noteworthy. Their deep learning model was able to extract clinically relevant insights by uncovering deeper, non-obvious patterns, likely mediated by confounding factors, even when direct physiological correlations are minimal [48].

Although none of the reviewed studies achieved clinically actionable lipid predictions, they collectively support the hypothesis that wearable-derived signals contain meaningful, non-invasive indicators of lipid physiology. The diversity of modeling approaches reflects the dynamic evolution of this research field and its ongoing refinement.

However, significant limitations remain. Wearable data are affected by sensor variability, user adherence and inconsistent long-term performance under real-world conditions [27, 49, 51]. Additional barriers include data privacy, lack of standardization and uncertainty regarding how wearable-derived metrics should inform clinical care [27, 49]. Integrating device- or ML-generated outputs into electronic health records also poses challenges, as current infrastructures are not designed for continuous or heterogeneous sensor data [51]. Regulatory approval requires extensive validation, yet most technologies reviewed have not undergone prospective, large-scale evaluation [27, 49, 51]. Data governance concerns, including security, patient consent, data ownership and GDPR compliance, further complicate implementation [27, 51].

Beyond technical considerations, patient acceptability and ethical implications remain central. Perceived intrusiveness, concerns about continuous monitoring and uncertainty regarding algorithm use may reduce acceptance, while frequent feedback could increase anxiety for some users [27]. Transparent communication about purpose, limitations and clinical relevance will be essential to maintain trust [27, 51].

Currently, the most predictive factor for CVD estimation using ML remains the input of a patient’s demographic data [38, 47]. However, even in this domain, the current evidence is insufficient and highlights the need for further refinement and validation [42–44]. Looking forward, interdisciplinary collaboration among engineers, clinicians, data scientists, and ethicists will be crucial to address the multifaceted challenges related to signal quality, model accuracy, data privacy and interoperability. Establishing clear clinical guidelines for interpreting wearable-derived metrics and standardizing data collection protocols will be essential steps toward ensuring safety and efficacy in these emerging technologies [32].

## Limitations

A limitation of this review is the time lag between the last literature search on 1 May 2024 and the submission of the manuscript. Given the high number of studies retrieved (14,863 records), subsequent screening, full-text evaluation, and data extraction required considerable time and effort. This process involved intensive and closely coordinated collaboration among four reviewers.

Although more recent studies may not have been captured, the comprehensive nature of the search and the robust methodology applied minimize the risk of missing significant developments. Future updates to this review may incorporate studies published after May 2024.

Another limitation is the restriction to English language publications, which may introduce language bias. This may particularly affect early feasibility studies or region-specific validation work less commonly available in English. As a result, the global representativeness of the evidence base may be reduced, and this limitation should be considered when interpreting the findings.

## Conclusion

Minimally and non-invasive sensing technologies and ML approaches offer a transformative opportunity for lipid profiling and cardiovascular risk assessment. While several prototypes and early clinical studies show strong potential, particularly in the integration with wearable systems, their routine application in preventive cardiology is still limited and requires further validation before clinical adoption.

ML has demonstrated its utility in cardiovascular risk stratification using conventional data, but lipid prediction based on wearable data is not yet mature enough for clinical decision-making. Future research should emphasize long-term, multimodal data collection, broader population validation, and clinical trials to assess the real-world effectiveness of these tools.

For gynecology, these technologies may be particularly relevant in the care of women during and after menopausal transition, when cardiovascular risk rises substantially due to hormonal and metabolic changes. By enabling accessible and non-invasive lipid monitoring, they could complement routine gynecologic follow-up, support early identification of high-risk patients, and strengthen tailored preventive strategies in this vulnerable patient group.

To advance this field, future research should prioritize standardized validation protocols, including harmonized reference procedures and external multi-center evaluation to ensure reproducibility across diverse populations and device types. Larger prospective and real-world studies are needed to assess long-term performance and define clinically meaningful thresholds for decision-making. Progress will depend on close collaboration between engineers, clinicians, data scientists, and regulatory experts to address challenges in signal quality, interoperability, and clinical integration. Such interdisciplinary efforts will be essential to translate promising prototypes into safe and broadly applicable tools for cardiovascular risk assessment.

## Supplementary Information

Below is the link to the electronic supplementary material.

Supplementary file1 Graphical Abstract (PDF 209 KB)

Supplementary file2 Search Strategy (PDF 108 KB)

Supplementary file3 PRISMA Flowchart (DOCX 57 KB)

Supplementary file4 Data Extraction Table (DOCX 61 KB)

## Data Availability

The data underlying this article are all available on the listed databases and are accessible through the references list.
