# Supplementary material for: Lipid monitoring using non-invasive measurement technologies and machine learning: a systematic review
Source: Arch Gynecol Obstet. 2026 Jan 30;313(1):71. doi: 10.1007/s00404-025-08254-6 (PMC12858588; doi:10.1007/s00404-025-08254-6)
Supplement: Supplementary file 1 — Supplementary file1 Graphical Abstract (PDF 209 KB) [file 404_2025_8254_MOESM1_ESM.pdf]

Lipid Monitoring Using Non-invasive Measurement technologies and Machine Learning: A systematic review

Background

- CVDs are the leading cause of death in women
- Lipid levels are a key biomarker for CVDs
- Conventional blood tests are invasive and inconvenient

Objective

To assess the availability, accuracy and clinical applicability of minimally and non-invasive lipid monitoring methods and Machine learning (ML)- based cardiovascular risk estimation

Methods

- Systematic search across databases for studies published between 2010 and 2024
- 14,863 records identified; 37 studies included
- Data extraction focused on wearable devices, measurement approach and predictive utility for cardiovascular outcomes

Key Findings

Innovative Methods for Lipid Monitoring

- Infrared/ Skin Sensors
- Saliva-based Sensors
- Smartphone-integrated Devices

Machine Learning & Wearables

- Pulse wave (PW) and Brachial-Ankle-PW-Velocity associated with Triglycerides (TG), LDL-Cholesterol, HDL-Cholesterol
- Resting heart rate associated with TG
- Deep learning-models for high Cholesterol prediction

Current Challenges

- Calibration issues, tissue variability, lack of standardisation of non-invasive sensors; most remain prototype stage
- Limited clinical validation of ML models
- Real-world use of wearables: sensor accuracy, data loss, user non-adherence, privacy concerns

Conclusion

Non-invasive sensing and Machine learning show strong potential for lipid profiling and cardiovascular risk assessment, but not yet clinically actionable; future work is needed such as clinical trials, long-term data, interdisciplinary collaboration, standardised protocols and Deep learning-model refinement
