# Supplementary material for: Lipid monitoring using non-invasive measurement technologies and machine learning: a systematic review
Source: Arch Gynecol Obstet. 2026 Jan 30;313(1):71. doi: 10.1007/s00404-025-08254-6 (PMC12858588; doi:10.1007/s00404-025-08254-6)
Supplement: Supplementary file 3 — Supplementary file3 PRISMA Flowchart (DOCX 57 KB) [file 404_2025_8254_MOESM3_ESM.docx]

**Identification of studies via databases and registers**

Records removed *before screening:*

Duplicate records removed (n=7,539 identified by Deduklick)

Duplicate records removed (n = 501: identified by Covidence n = 492; Duplicates identified manually n = 9)

Records identified from*:

Total number: (n= 22,903)

Medline (n:3,553)

Embase (n=6,270)

Cochrane Library (n=1,084)

WOS (n=5,097)

Scopus (n=5,885)

Clinical trials.gov (N=1,014)

**Identification**

Records screened

(n = 14,863)

Records excluded**

(n = 13454)

Reports sought for retrieval

(n = 1,409)

Reports not retrieved

(n = 118)

**Screening**

Reports excluded (non-lipid related reasons)

Wrong Topic (n = 623)

Wrong Language (n =13)

Wrong Indication (n = 4)

Wrong Method (n = 210)

Wrong Intervention (n = 34)

Wrong Outcomes (n = 38)

Wrong Study Design (n = 65)

Duplicate (n = 1)

Animal Testing (n = 1)

Wrong Population (n = 7)

Lack of Quality (n = 6)

Reports excluded (blood pressure-related (n=252)

Reports assessed for eligibility

(n = 1,291)

Studies included in review

(n=37)

**Included**

*Consider, if feasible to do so, reporting the number of records identified from each database or register searched (rather than the total number across all databases/registers).

**If automation tools were used, indicate how many records were excluded by a human and how many were excluded by automation tools.

Source: Page MJ, et al. BMJ 2021;372:n71. doi: 10.1136/bmj.n71.

This work is licensed under CC BY 4.0. To view a copy of this license, visit <https://creativecommons.org/licenses/by/4.0/>
