# Supplementary material for: Lipid monitoring using non-invasive measurement technologies and machine learning: a systematic review
Source: Arch Gynecol Obstet. 2026 Jan 30;313(1):71. doi: 10.1007/s00404-025-08254-6 (PMC12858588; doi:10.1007/s00404-025-08254-6)
Supplement: Supplementary file 4 — Supplementary file4 Data Extraction Table (DOCX 61 KB) [file 404_2025_8254_MOESM4_ESM.docx]

# Data Extraction Table

| **Nr.** | **PMID** | **Title** | **Author, Year** | **Study design** | **Sample size** | **Methods** | **Intervention** | **Results** | **Outcome/Discussion** |
| --- | --- | --- | --- | --- | --- | --- | --- | --- | --- |
| 1 | DOI: 10.1109/ICOIACT55506.2022.9972007 | A Non-invasive Cholesterol Measuring Device using a Photodiode Sensor with a BLYNK Interface | Nur Hasanah Ahniar et al., 2022 | Experimental Study | 5 participants, aged 22-25 | Development and experimental testing of a non-invasive cholesterol measuring device using a photodiode sensor with a BLYNK interface for data display and possibly remote monitoring.  Designing block diagrams, power supply circuits, light source circuits, photoresistor transducer circuits, Arduino microcontrollers, and software on mobile phones are all part of the design and construction of a cholesterol measurement device. Used a photodiode sensor to detect infrared light absorption in body tissues. Employs a Near-Infrared (NIR) sensor with an infrared (IR) light emitting diode (LED) 333-A Ever light emitter (940nm wavelength). The detector used is a PT 333-3C Ever light photodiode (spectral bandwidth of 400nm - 1100nm). | First, a literature review was made for detecting and describing methods to measure cholesterol non-invasively. Then, an experimental comparison of the non-invasive photodiode sensor device with gold-standard invasive measurement methods, before and after meals. | Average error: 1.46%, accuracy: 98.54% in comparison to the invasive standard method, strong correlation R^2^ = 0.9878 | The accuracy matches the standard measuring method; improved user comfort; less medical waste; a higher intensity LED for wider application is recommended |
| 2 | DOI: 10.1109/IC2IE56416.2022.9970087 | Non-invasive IoT Home Medical Check-up Programming to Monitor Blood-Sugar, Cholesterol, Uric-Acid and Body Temperature | Helmy Yusuf Darmawan et al., 2022 | Experimental study | Not explicitly mentioned; multiple healthy subjects | Use of ESP32 WEMOS LOLIN 32 Lite microcontroller, MAX30105 sensor (for blood sugar, cholesterol, uric acid (UA)), and MLX90614 sensor (for temperature). Data transmitted to a PostgreSQL database and displayed on an Android app; calibration to reference device; continuous data logging and cloud storage. | Experimental study focusing on the development and testing of a non-invasive multiparametric Internet of Things (IoT) home medical check-up tool: simultaneous measurements (finger placement) for cholesterol, UA, blood sugar and body temperature. | Accuracy rates: Blood sugar (91%), Cholesterol (91%), UA (82%), fast data transmission (<0.1 s delay). | Demonstrates practical potential for remote, non-invasive home screening. Needs further calibration for clinical use. |
| 3 | PMID: 30010594    DOI: 10.1109/TBCAS.2018.2845856 | Blood Cholesterol Monitoring with Smartphone as Miniaturized Electrochemical Analyzer for Cardiovascular Disease Prevention | Yusheng Fu et al., 2018 | Experimental study | 126 healthy patients | Comparison between plug-in smartphone electrochemical (EC) analyser and clinical standard device: An EC analyser is integrated into a smartphone to measure the current generated by the enzymatic reaction with a total cholesterol (TC) test strip. The disposable test strip converts the biochemical signal to an electrical signal through an EC reaction. The EC module is composed of an embedded circuit for signal processing and communication with the smartphone. The system uses 1 μL of finger-pricked whole blood. The concentration of TC is sensed by a printed carbon electrode after an EC reaction under a specialised enzyme. The generated EC current is acquired by an analog-to-digital converter (ADC) and analysed by the microcontroller unit (MCU) of the module. Data is communicated through inter-integrated circuit (IIC), saved, and uploaded to a personal health management center through the internet. | Validation study with patient samples; development and testing of a smartphone-based EC analyser for blood cholesterol monitoring. | High correlation to clinical analyser (R² = 0.997); reproducibility (coefficient of variation (CV) 2–4%); valid for point-of-care use | The proposed TC test strip was relatively stable and has good reproducibility. The three tested chronoamperometric curves were very close, demonstrating stability. The system can provide accurate evaluation of a patient’s blood lipid level as compared to a clinical biochemical analyser. The proposed medical smartphone system is a promising platform as a point-of-care device for blood TC monitoring. |
| 4 | PMID: 29994232    DOI: 10.1109/JBHI.2018.2845860 | Blood Triglyceride Monitoring with Smartphone as Electrochemical Analyzer for Cardiovascular Disease Prevention | Jian Wang et al., 2019 | Experimental Study | 106 hyperlipidaemia patients | Development and testing of a smartphone-integrated EC analyser; uses disposable EC blood triglyceride (TG) test strips (1–2 μL finger-pricked blood); TG sensory module includes a MCU and peripheral circuit for signal processing; The module provides the electrical voltage for the test strip to activate the EC reaction, and the generated EC current is acquired and mapped into the concentration of blood TG. Finger blood is applied directly on the test strip by capillary force. The point-of-care TG level is stored in an online personal health center updated by the smartphone. | Miniaturised smartphone point-of-care (POC) device for monitoring blood TG and TC; tested and compared to the clinical gold standard analyser; reproducibility/CV and calibration range assessed | Blood TG: highly linear correlation to clinical analyser (R²=0.989 for TG); reproducibility: CV low (e.g. ~2–4%); minimum TG diff.: 0.01 mmol/L; similar approach used for TC monitoring; accuracy validated on 106 samples | The developed medical smartphone provides a point-of-care analytical device for blood TC and TG monitoring; rapid, home-based monitoring similar to the hospital analyser; supports patient-managed chronic care; highlights benefit for mobile health |
| 5 | PMID: 24336861    DOI: 10.1039/c3lc51194d | Cholesterol testing on a smartphone | Vlad Oncescu et al., 2014 | Experimental study | 9 volunteers | A smartphone accessory (smartCARD ) attaches to a smartphone; with standard colorimetric test strips; the system utilises the smartphone's flash with a light diffuser to illuminate the strip. An iOS app is developed to analyse the colour reaction, quantify cholesterol levels, and display the results. The app uses a calibration curve linking cholesterol concentration to the HSL (Hue, Saturation, Lightness) colour values extracted from the image of the test strip. The algorithm includes background shift correction and verification of test validity based on hue values. The data was compared to CardioCheck PA. | Development and testing of a smartphone accessory (smartCARD) and software application for quantifying cholesterol levels in blood using standard test strips with single blood drops. | Deviation ≤5.5% from CardioChek PA; inter-phone difference <5.8%; per-test accuracy typically <1.8%. | The system can accurately quantify TC levels in blood within 60 seconds by imaging standard test strips. Technology improves traceability/robustness over home kits; enables error flagging; discussed future improvement for calibration. |
| 6 | PMID: 31820750    DOI: 10.1039/c9an01679a | Sensitive and non-invasive cholesterol determination in saliva via optimization of enzyme loading and platinum nano-cluster composition | Kyu Shik Eom et al., 2020 | Experimental study | 3 hyperlipidaemia patients | Fabrication of a simple, disposable, enzyme-based EC biosensor. Optimisation of the vertical structure of platinum nanoclusters (Pt-NC). Immobilisation of an appropriate volume of enzyme. Measurement of cholesterol levels in saliva using the fabricated biosensor. EC measurements using cyclic voltammetry and chronoamperometry. | Development and testing of a disposable, enzyme-based EC biosensor for detecting cholesterol in saliva. | Linear range: 2 to 486 μM. Limit of detection: approximately 2 μM. - Sensitivity: 132 μA mM−1 cm−2. Good specificity for ascorbic acid, UA, dopamine, glucose, and lactate. Cholesterol levels measured in the saliva of three patients with hyperlipidaemia were 520, 460, and 290 μM. | Pt-NC based enzyme sensor is a promising candidate for the detection of cholesterol in human saliva; broader clinical testing is recommended. |
| 7 | DOI: 10.1016/j.snb.2022.133214 | All-in-one microfluidic device with an integrated porous filtration membrane for on-site detection of multiple salivary biomarkers | Rajendran Vinoth et al., 2023 | Experimental study | Not explicitly mentioned: Saliva samples were collected from healthy volunteers. Glucose levels were tested in saliva from three healthy individuals before and after consuming glucose-rich drinks. | 3D-printed microfluidic device with integrated porous filtration membrane for multiplexed detection of salivary biomarkers (glucose, lactate, cholesterol, and UA): The device uses enzyme-based EC sensors integrated into a 3D-printed platform. A ‘channel-inside-channel’ configuration was used to facilitate passive saliva flow and prevent clogging. Flexible porous filtration membranes were incorporated to eliminate biofouling caused by salivary proteins. The system includes wireless data collection via a MATLAB interface. Biomarkers were measured using amperometric techniques, and results were validated against high-performance liquid chromatography (HPLC) and colorimetric assay methods. | Developing and testing of a point-of-care, non-invasive salivary microfluidic sensor, multiplexed for 4 biomarkers | The device demonstrated good agreement with HPLC and commercial enzymatic assay methods for glucose, with an average Pearson correlation coefficient of 0.9161 compared to finger-prick blood glucose measurements. Saliva cholesterol detected <15 μM; repeatable, reliable, correlated with HPLC; usable for >4 h; stable and consistent | The system successfully differentiated glucose levels in saliva before and after consuming glucose-rich drinks. The microfluidic platform is effective for multiplexed biomarker detection and point-of-care analysis. |
| 8 | DOI: 10.1609/aaai.v32i1.11891 | DeepHeart: Semi-Supervised Sequence Learning for Cardiovascular Risk Prediction | Brandon Ballinger et al., 2018 | Experimental ML model development study | 14,011 participants (recruited via an Apple Watch app, contributing 57,675 person-weeks of data. Specific labelled data includes: 2,331 participants with high cholesterol, 2,230 with hypertension, 1,016 with sleep apnoea, and 462 with diabetes.) | Semi-supervised sequence learning and heuristic pretraining methods were used to train the long short-term memory (LSTM) model. Data was collected from wearable heart rate (HR) sensors paired with step count data. Diagnoses (e.g., high cholesterol) were derived from validated health surveys and medical history. The LSTM model architecture included temporal convolutions and bidirectional LSTM layers for multi-task predictions. Pretraining approaches included unsupervised sequence learning (autoencoder) and heuristic pretraining based on HR variability metrics. | Development and validation of a semi-supervised, multi-task LSTM model for predicting cardiovascular risk factors using wearable HR data and self-reported health survey. No physical intervention; passive monitoring with wearables (Fitbit, Apple Watch, Android). | The model achieved high accuracy for detecting various conditions: diabetes (area under the curve (AUC) = 0.8451), high cholesterol (AUC = 0.7441), hypertension (AUC = 0.8086), and sleep apnoea (AUC = 0.8298). | Outperformed traditional machine learning (ML) models (e.g., logistic regression, support vector machine (SVMs)) trained on hand-engineered biomarkers. Heuristic pretraining improved performance for high cholesterol prediction but showed mixed results for other conditions. Demonstrated feasibility of using consumer-grade wearables for cardiovascular risk prediction. Demonstrated feasibility of cardiovascular risk stratification from consumer wearables via ML; utility for mobile public health screening. |
| 9 | PMID: 35904853  PMCID: PMC9377462  DOI: 10.2196/34669 | High-Resolution Digital Phenotypes from Consumer Wearables and Their Applications in Machine Learning of Cardiometabolic Risk Markers: Cohort Study | Weizhuang Zhou et al., 2022 | Cross-sectional observational cohort Study | 692 healthy volunteers from the SingHEART study | Collected HR and step count data from Fitbit Charge HR devices over 3-5 days. Used a framework to extract 66 high-resolution digital phenotypes from wearable data, encoding contextual information about physiological states. Applied ML models to relate these phenotypes to clinical and genomic risk markers for blood pressure (BP), lipid, weight, and sugar abnormalities. | Observational cohort study using ML to analyse wearable data and predict cardiometabolic risk markers. | High-resolution features had higher predictive value than typical baselines (age, gender, resting HR) for clinical markers of cardiometabolic disease risk (17.9% and 7.36% improvement in Brier score, P<.001). HR dynamics in sedentary states were most predictive of lipid abnormalities and obesity, while active states were more predictive of BP abnormalities (P<.001). High-resolution patterns in wearable HR recordings better represented subtle physiological dynamics related to genomic risk for cardiometabolic disease (11.9%-22.0% improvement in Brier scores; P<.001). | Advanced feature extraction from wearables enables better individualised risk scoring, capturing subtle genetic and metabolic risk signals. |
| 10 | PMID: 29485983  PMCID: PMC5828350  DOI: 10.1371/journal.pbio.2004285 | Beyond fitness tracking: The use of consumer-grade wearable data from normal volunteers in cardiovascular and lipidomics research | Weng Khong Lim et al., 2018 | Prospective observational cohort study | 233 healthy volunteers | Tracked subjects using a Fitbit Charge HR. Collected accelerometery and HR measurements, lifestyle questionnaires, clinical measurements (weight, height, waist circumference, BP, etc.), lipid panel values, blood glucose test, cardiac magnetic resonance imaging (CMR), and lipidomic profiling. Performed integrative analysis of the dataset. | Observational study integrating wearable data with other clinical and lifestyle data. | Subjects can be stratified into distinct clusters based on daily activity patterns, which are marked by distinct demographic and behavioural patterns. Resting HRs (RHRs) performed better than step counts in being associated with cardiovascular and metabolic disease markers. Step counts identified relationships between physical activity and cardiac remodelling. Wearable-derived activity levels can be used to identify known and novel activity-modulated sphingolipids that are in turn associated with insulin sensitivity. | Wearable data can serve as a practical cardiovascular/metabolic risk marker and adjunct for personalised prevention. Limits were short tracking windows. |
| 11 | PMID: 32419802  PMCID: PMC7210560  DOI: 10.1155/2020/3926851 | Radial Pulse Wave Signals Combined with Ba-PWV for the Risk Prediction of Hypertension and the Monitoring of Its Accompanying Metabolic Risk Factors | Zhen Qi et al., 2020 | Observational cohort study | 523 adults, 264 hypertensive, 259 normotensive | Radial artery pulse wave signals recorded using a DDMX-100 pulse measurement device. Measurement of brachial-ankle pulse wave velocity (Ba-PWV). Collection of data on metabolic risk factors (fasting plasma glucose (FPG), TC, TG, High-Density Lipoprotein Cholesterol (HDL-C), Low-Density Lipoprotein Cholesterol (LDL-C), UA). Linear and stepwise regression analysis to assess associations. Calculation of AUC, Net Reclassification Improvement (NRI), and Integrated Discrimination Improvement (IDI) to compare discriminative ability among models. | Observational cohort study with regression analysis and risk assessment; no treatment/intervention, wrist-worn pulse measurements and Ba-PWV, compared to clinical risk factors. | Radial artery pulse wave variable h3/h1 selected as a sensitive influential factor for BP. A new model with h3/h1 had a higher AUC than the reference model (0.86 vs 0.84; P = 0.030). NRI and IDI for the new model were 50.0% (P = 0.017) and 3.16% (P = 0.044), respectively. Decrease of t4, t5, and h5 associated with higher FPG, TC, LDL-C, and UA and lower HDL-C. | Pulse wave analysis enhances hypertension and metabolic risk stratification; supports use of wearable pulses for remote risk evaluation; cross-sectional/convenience sample limits causal inference |
| 12 | PMID: 33145438  PMCID: PMC7603302  DOI: 10.1038/s41746-020-00349-5 | Performance and clinical utility of supervised machine-learning approaches in detecting familial hypercholesterolaemia in primary care | Ralph K. Akyea et al., 2020 | Retrospective cohort study | 4,027,775 individuals with TC measured from 1 January 1999 to 25 June 2019 | Used five ML algorithms (logistic regression, random forest (RF), gradient boosting machines, neural networks, and ensemble learning) to detect familial hypercholesterolaemia (FH) in primary care clinical records. | No physical/behavioural intervention; analytic study of ML for undiagnosed hypercholesterolaemia. | All ML approaches apart from logistic regression (AUC, 0.81) had high predictive accuracy (AUC > 0.89). The clinical case-finding workload required for yield of cases differed substantially between models. Ensemble learning exhibited a dominant positive likelihood ratio (45.5) compared to all other ML models (7.0–14.4). | ML can dramatically improve case-finding for FH and inherited lipid disorders; clinical trade-offs between yield and workload depending on model; supports digital screening/risk stratification at scale |
| 13 | PMID: 37557962  PMCID: PMC11233111  DOI: 10.1016/j.bbi.2023.08.008 | Wearable technologies for health research: Opportunities, limitations, and practical and conceptual considerations | Lydia G. Roos, George M. Slavich 2023 | Systematic literature review | - | Literature review, critical evaluation of device validation, cross-study comparability, sampling rates, emphasis on cardiovascular and stress/behavioural research. | Discusses the use of wearable devices for high-frequency, non-invasive monitoring of physiological and behavioural processes in real-world settings. Highlights key metrics derived from wearables, such as HR, HR variability, BP, respiration rate, sleep patterns, physical activity, and stress-related electrodermal activity (EDA). Explores the integration of wearable data with ML to develop digital biomarkers for health monitoring. Provides practical guidance for researchers on device selection, data quality considerations, and participant usability issues. | Wearables enable scalable and continuous monitoring of physiological states in naturalistic environments, bridging the gap between laboratory-based research and real-world health interventions, with wide potential for phenotyping CVDs and stress. | Stress tracking features in wearables have potential but require further validation. Key limitations include variability in device accuracy across metrics and challenges with data sampling rates and participant adherence, device/data validity, lack of standardization and statistical complexity. Provides a framework of 10 key questions to guide researchers in designing wearable-based studies effectively. |
| 14 | PMID: 36679626  PMCID: PMC9865666  DOI: 10.3390/s23020828 | Smart wearables for the detection of cardiovascular diseases | Mohammad Moshawrab et al., 2023 | Systematic literature review | 87 peer-reviewed studies included (from 4002 initial) | Followed PRISMA guidelines to search IEEE, PubMed, and Scopus databases. Analysed studies based on publication year, vital signs recorded, diseases studied, hardware used, smart models applied, datasets utilised, and performance metrics reported. | Review of interventions studied in included works; mainly monitoring and diagnostics using wearable electrocardiogram (ECG)/Photoplethysmography (PPG), wristbands, patches, smart clothing and smartphone integration. Focused on the role of artificial intelligence (AI) and Information and Communication Technology (ICT) in enhancing wearable devices for CVD management. | Smart wearables are effective in detecting, predicting, and managing cardiovascular diseases (CVDs) with high accuracy. Across studies, ML-powered wearables detect/or predict atrial fibrillation (AF), arrhythmias, myocardial infarction with high accuracy—usually >90%, many report sensitivity/specificity ~95–99%. | Interest in smart wearables for CVD management has increased significantly over the years. Challenges include frequent small sample sizes, dependency on open datasets, and lack of external/clinical validation. Future research should focus on real-world integration, privacy/data security, robust benchmarking, and application to larger/ higher-risk populations. |
| 15 | PMID: 36862812  PMCID: PMC9991078  DOI: 10.1161/CIRCRESAHA.122.322389 | Wearable Devices in Cardiovascular Medicine | Andrew Hughes et al., 2023 | Systematic literature review | - | Summarises technical features of wearables and different ML techniques for analysing wearable data. Reviews clinical applications of wearables in arrhythmia screening, heart failure (HF) management, and peripheral artery disease monitoring. Identifies challenges such as device accuracy, data privacy concerns, regulatory hurdles, and lack of clinical staff to interpret wearable data. | Discusses wearable devices such as smartwatches, activity trackers, patches, rings, and clothing-embedded devices. Explores sensor technologies like accelerometery, PPG, ECG), seismocardiogram, ballistocardiogram, and continuous glucose monitoring (CGM). Highlights ML techniques (supervised, unsupervised, semi-supervised, reinforcement learning, and deep learning (DL)) for analysing wearable data. | Wearables increase steps (~1800/day), modestly reduce weight, improve AF detection (PPV 0.84–0.92), facilitate some improvements in HF, support diabetes control; the best outcomes were with device plus coaching. | Wearable devices provide continuous monitoring of physiological parameters like HR, rhythm, BP, oxygen saturation, sleep patterns, and physical activity. ML enhances the clinical utility of wearables by enabling prediction and classification tasks for CVDs. Challenges include variability in device accuracy across metrics and difficulties in integrating wearables into routine clinical practice. Proposed solutions include improving device accuracy, addressing privacy concerns, creating reimbursement policies, and training dedicated clinical staff for wearable data interpretation. |
| 16 | DOI: 10.1063/5.0108884 | Machine Learning Approach for Heart Disease Prediction: A Survey | S. Gupta, P. Sharma, 2022 | Systematic literature review | - | The study reviews and analyses different ML techniques applied to heart disease prediction. The review includes discussion of data mining, feature selection algorithms and common classifier models. | The interventions examined include Pre-processing techniques: Methods for cleaning and preparing the data, such as handling missing values and scaling features. Feature extraction methods: Approaches for selecting the most relevant features from the dataset to improve model performance, including Minimum Redundancy Maximum Relevance (MRMR), Least Absolute Shrinkage and Selection Operator (LASSO) Algorithm, and Log-Likelihood Based Forward Selection (LLBFS). Classification Algorithms: The use of various supervised ML algorithms such as Artificial Neural Networks (ANN), Support Vector Machines (SVM), Decision Trees (DT), and K-Nearest Neighbors (KNN) for predicting heart disease. | The study highlights the importance of each step in the heart disease prediction process, including data pre-processing, feature extraction, and the selection of appropriate classification algorithms. The techniques provided an accuracy up to 96.3% for predicting heart disease. | The main finding of the survey is that supervised ML algorithms are the most efficient for predicting cardiovascular disorders. It highlights that supervised learning methods, which learn from labelled data, generally outperform other approaches in this context. ML can support clinicians in reducing misdiagnosis and healthcare costs. Challenges remain with data quality. |
| 17 | DOI: 10.3390/healthcare11162240 | A Systematic Review of Machine Learning and IoT Applied to the Prediction and Monitoring of Cardiovascular Diseases | Alejandra Cuevas-Chàvez et al., 2023 | Systematic literature review | 164 papers included | 164 journal papers: 82 proposals for CVD detection using IoT/ Internet of Medical Things (IoMT) technologies and 85 proposals for CVD using ML techniques. PRISMA-guided systematic review. Databases: PubMed, IEEE Library, SpringerLink, ScienceDirect. Studies (2016-2023) focusing on IoT/IoMT and ML for CVD detection/prediction/monitoring. PICOC framework applied. | The systematic review highlights IoT/IoMT technologies, ML techniques, datasets, and the most discussed CVDs to detect, predict or monitor CVDs in various populations, focusing on evaluation metrics, relevant datasets, and technological trends. | Neural networks have been popularly used, achieving an accuracy of over 90%, followed by RF, XGBoost, KNN, and SVM. | IoT/IoMT technologies show strong capacity for predicting CVDs in real time, ensemble techniques obtained one of the best performances in the accuracy metric, and hypertension and arrhythmia were the most discussed diseases. Finally, one of the main obstacles for ML approaches for CVD prediction is the lack of public data. |
| 18 | DOI: 10.1007/s12551-022-01040-7 | Recent developments in modelling, imaging, and monitoring of cardiovascular diseases using machine learning | Hamed Moradi et al., 2023 | Systematic literature review | - | Discusses the utility of ML techniques, in particular DL methods, to overcome the limitations of computational fluid dynamics, blood flow imaging, and accurate detection of CVDs using data collected by wearable sensors. | Synthesis of ML (incl. DL etc) into computational fluid dynamics (CFD), blood-flow imaging (4D-MRI), and wearables for CVD monitoring. | DL can accelerate computational models (e.g. CFD), enhance image resolution/denoising in MRI, and accurately classify CVDs from wearable sensor data. Specific case studies indicate up to 600-fold speed improvement, and AUC/accuracy in clinical prediction >90% in modern studies. | ML techniques, especially DL and Physics-Informed Neural Networks (PINNs) can accelerate flow modelling, enhance the resolution while reducing noise and scanning time of current blood flow imaging techniques, and accurately detect CVDs using data collected by wearable sensors. Such models can be further improved by incorporating fluid–structure-interaction models to predict  the deformation of blood vessels under pulsatile flows,  movement of blood cells inside the vessels, as well as the  complex dynamics of blood cells at the vicinity of heart  valves. |
| 19 | DOI: 10.3390/electronics12071558 | Reviewing Multimodal Machine Learning and Its Use in Cardiovascular Diseases Detection | Mohammad Moshawrab et al., 2023 | Systematic literature review | 13 key studies reviewed | Technical literature review of multimodal ML definitions, frameworks, data fusion approaches, and CVD studies. | The authors reviewed multimodal ML from various angles, including technical definitions, differences from other domains, such as classical ML, ensemble ML and others, available frameworks, and other details. They listed the state of the art of the use of Multimodal ML technology in CVD detection and its prediction and discussed the technical details in the reviewed literature. Later, challenges that hinder progress in this field are discussed and therefore, some future prospectives that could help overcome these challenges are proposed. | Most multimodal ML models for CVDs outperform or match classical ML in accuracy/AUC (e.g. 94.8%+ accuracy, AUC up to 0.95). Late/intermediate fusion and hybrid architectures are frequent, integrating Electronic Health Records (EHR), imaging, wearables, or clinical metadata. | Multimodal ML is a new technique that enables the simultaneous use of multiple models and data types in the creation of complex ML and DL models. Multi modal ML has the potential to significantly improve the accuracy and effectiveness of AI applications, especially in healthcare, where it has already become an important part of everyday patient care by addressing the problem of data heterogeneity. In particular, the technical features of Multimodal ML, such as data fusion and workflows, were covered, and the differences with other technologies, such as Ensemble Learning, were highlighted. In addition, an overview of the application of Multimodal ML in the diagnosis and prediction of CVDs was provided, highlighting the encouraging results to date and the room for growth in this area. Privacy, bias, and interpretability of results are just some of the remaining difficulties that need to be addressed, as with any rapidly evolving technology. However, it is likely that these obstacles can be addressed through further research and development and that multimodal ML will continue to play an important role in the development of AI applications in a variety of sectors, particularly healthcare. |
| 20 | DOI: 10.1016/j.rico.2023.100362 | Learned prediction of cholesterol and glucose using ARIMA and LSTM models – A comparison | Umapathi Krishnamoorthy et al., 2024 | Experimental study | 1 patient | Development of a hardware prototype for non-invasive glucose and cholesterol measurement using NIR sensors. The data collected was used to train and compare the prediction efficiency of the Auto-Regressive Integrated Moving Average (ARIMA) model and the LSTM model. | Non-invasive cloud-based monitoring and real-time prediction of glucose and cholesterol using ARIMA and LSTM. | The ARIMA model surpasses the LSTM model in predicting glucose and cholesterol levels, supported by a lower Root Mean Square Error (RMSE) of the ARIMA model (about 71.7% less for Glucose and 50.3% less for Cholesterol) compared to the LSTM model, indicating higher prediction accuracy. | ARIMA outperforms LSTM for forecasting in this application. Combined with a non-invasive device, this approach could improve early diagnosis and monitoring of diabetes and CVD risk. |
| 21 | DOI: 10.1007/s12170-023-00731-4 | Artificial Intelligence for Risk Assessment on Primary Prevention of Coronary Artery Disease | Shang-Fu Chen et al., 2023 | Systematic literature review | - | Summarises recent advances in AI for risk assessment of primary prevention of coronary artery disease (CAD), focusing on modelling approaches and application to multi-modal data. | This article reviews how advances in big data and predictive modelling foreshadow a promising future of improved risk assessment and precision medicine for CAD. AI could be used with different diagnostic tools such as: Laboratory Biomarkers, Genetically Informed Risk Assessment Models, Multi-omics Data, Real-Time Sensor-Based Risk Monitoring, Heart Rhythm Monitoring via different sensors, Physical Activity Monitoring, Biochemical Sensors, Environmental Sensing and Advanced Application in Non-Invasive Imaging for CAD Risk evaluation such as Coronary Artery Calcium Scoring, Coronary CT Angiography, Nuclear Imaging and Retinal Imaging. | AI/ML models, especially ensemble and neural networks, outperform traditional risk scores; multi-modal risk models incorporating biomarkers, genetics and imaging show further promise, but clinical translation and validation are still lacking. | AI offers a multi-modal risk prediction using standard biomarkers, genetic and other omics technologies, a variety of biosensors, and unstructured data from EHRs). However, gaps remain in clinical validation of AI models, most notably in the actionability of complex risk prediction for more precise therapeutic interventions. |
| 22 | DOI: 10.1038/s41598-021-04649-y | Application of ensemble machine learning algorithms on lifestyle factors and wearables for cardiovascular risk prediction | Weiting Huang et al., 2022 | Prospective observational study with ML development and validation | 600 Participants aged 21 to 69 years free of CVD. Subset analysis for activity tracker data was performed on 430 of the 600 volunteers who had adequate data. | Collected data from lifestyle questionnaires, clinical blood tests, 24-h ambulatory BP and HR monitoring, and activity tracking data (Fitbit Charge HR). Used ensemble MLAs based on naive bayes, RF, and support vector classifier for low-risk categories, generalised linear regression, support vector regressor, and stochastic gradient descent regressor for high-risk categories. Compared the MLAs against the Framingham Risk Score (FRS). Stratified subjects into low risk (calcium score 0) and high risk (calcium score ≥ 100) based on coronary artery calcium scoring. | Comparison of ensemble ML models utilising surveys, bloods, BP, wearable data to standard risk prediction models. | All MLAs outperformed the FRS for both low and high-risk categories. MLA based on the lifestyle questionnaire alone achieved an AUC of 0.715 (95% CI 0.681, 0.750) and 0.710 (95% CI 0.653, 0.766) for low and high risk, respectively. Combining all groups of risk factors (lifestyle survey questionnaires, clinical blood tests, 24-h ambulatory BP and HR monitoring) along with feature selection, the prediction of low and high CVD risk groups was further enhanced to 0.791 (95% CI 0.759, 0.822) and 0.790 (95% CI 0.745, 0.836). Self-reported physical activity, average daily HR, awake BP variability, and percentage of time in diastolic hypertension were important contributors to CVD risk classification. | Ensemble ML incorporating lifestyle, clinical and wearable data can improve individualised CVD risk stratification beyond conventional algorithms. |
| 23 | DOI: 10.1371/journal.pone.0213653 | Cardiovascular disease risk prediction using automated machine learning: A prospective study of 423,604 UK Biobank participants | Ahmed M. Alaa et al., 2019 | Prospective cohort study | 423,604 adults (UK Biobank) | Compared performance of “AutoPrognosis” (automated ML pipeline selecting, tuning, and ensemble models) to Framingham and Cox models; used 473 variables; primary endpoint: 5-year CVD events. | Compared the ML-based multi-modal risk prediction versus standard risk scores in large general population. | AutoPrognosis improved risk prediction (AUC 0.774) compared to Framingham (AUC 0.724); inclusion of non-traditional variables (e.g., walking pace, health perception) picked up by ML as relevant predictors. | Automated ML using diverse variables achieves better CVD risk prediction than standard models—particularly in population subgroups—thus supporting wider adoption for prevention strategies. |
| 24 | DOI: 10.3390/pr10040749 | Predictive Classifier for Cardiovascular Disease Based on Stacking Model Fusion | Jimin Liu et al., 2022 | Cross-sectional model development study | 918 patient records (Heart Dataset); 303 (Heart Attack Dataset) | Developed a stacking model combining various ML technologies such as RF, Gradient Boosting, and Logistic Regression on public datasets of clinical/biochemical/lifestyle CVD risk features and compared various feature selection strategies. | Stacked ML algorithms for early prediction of CVD (feature inputs include BP, cholesterol, age, etc.); tested on general medical datasets. | The stacking model outperformed individual models with an AUC of 0.927 and accuracy of 88.1%, showing strong predictive power for CVD classification. | Multi-parametric data significantly improve predictive accuracy for CVDs; this enables better stratification, early intervention, and could inform clinical decision support and POC risk prediction tools. |
| 25 | PMID: 36140511 PMCID: PMC9498278 DOI: 10.3390/diagnostics12092110 | Smart Consumer Wearables as Digital Diagnostic Tools: A Review | Shweta Chakrabarti et al., 2022 | Systematic literature review | - | Review of literature on the use of smart consumer wearables as digital diagnostic tools for CVD, neurological, metabolic, sleep and psychological disorders with a focus on ML. | Applications of sensors and ML in consumer-grade wearables for digital diagnosis. | Wearable devices can collect different data as step counts, HR, sleep duration, calories burnt, stress and oxygen levels through different types of sensors. HR, step count and energy consumed have apparently been associated with cardiovascular disorders by researchers. In detail, the authors pointed Apple Watches out, that can be useful in the detection of several CVDs such as myocardial ischemia or cardiac arrhythmias by collecting HR data, such as “Fitbit Charge HR” which’s Sleep tracking acts as a CVD marker. From a data analysis of a multi-modal phenotype, it was found that those parameters derived from wearables had an association with markers of CVDs, such as waist circumference and body mass index. Lipids itself were not described in this study. | Wearables offer promising potential for real-time, personalised diagnostics, especially for CVD, but challenges remain regarding data quality, security and regulation. |
| 26 | DOI: 10.1109/ACCESS.2023.3236002 | MedAi: A Smartwatch-Based Application Framework for the Prediction of Common Diseases Using Machine Learning | Shinthi T. Himi et al., 2023 | Experimental study | 278 patient records | Development of a smartwatch ("Sense O'Clock") equipped with eleven sensors, a ML model for data analysis, and a mobile application for displaying prediction results.  Multiple ML algorithms (RF, SVM, KNN, XGBoost, LSTM) compared. | On-device ML prediction of 12 diseases, including ischaemic heart disease and hypertension, using biosensor data. | RF algorithm outperformed other ML algorithms (SVM, KNN, XGBoost, LSTM) with an accuracy of 99.4% in predicting diseases such as ischemic heart disease, hypertension, respiratory disease, hyperthyroidism, hypothyroidism, stroke, myocardial infarction, kidney failure, gallstones, diabetes, and dyslipidaemia. | Smartwatches with multisensor arrays and robust ML models (especially RF) enable highly accurate, early and multi-disease prediction for preventive digital health. |
| 27 | DOI: 10.3390/bios11070228 | Building a Cardiovascular Disease Prediction Model for Smartwatch Users Using Machine Learning: Based on the Korea National Health and Nutrition Examination Survey | Min-Jeong Kim et al., 2021 | Cross-sectional model development study | 6’170 adults | Developed a method to predict CVDs by using health data measured by Samsung Smartwatches. Health data of 6’170 participants was analysed, after removing missing and abnormal values. The authors used different ML techniques such as logistic regression, artificial neural network and support vector machine after selecting the input variables. The selected input data was based on the Samsung Smartwatch’s capabilities in measuring health data, such as demographic data, systolic and diastolic BP, fasting blood glucose, pulse rate, perceived stress level and body mass index. | The model was used to predict the prevalence of the following cardiovascular—related conditions: Hypertension, Dyslipidaemia, Stroke, Myocardial infarction, Angina pectoris and Diabetes. The authors did not use specific lipid profile data as input variables but instead relied on more general health indicators as described before. The inclusion of dyslipidaemia in the predicted conditions was based on its role as a major risk factor for CVDs, rather than being specifically predicted by using lipid-related measurements. | The Gradient Boosting Machine performed best among the models: Accuray 0.874, AUC: 0.850, Sensitivity: 0.788 and Specificity: 0.882. | The study concludes that it is feasible to build a practical CVD prediction model using features available from smartwatches and user-reported data, potentially enabling early screening and personalized health monitoring. |
| 28 | PMID: 37806179 DOI: 10.1016/j.ijmedinf.2023.105218 | Use of wearables for monitoring cardiometabolic health: A systematic review | Mikyoung A. Lee et al., 2023 | Systematic Literature Review | 53 Studies | Systematic review of empirical studies (2016–2021) on wearables for cardiometabolic diseases; assessment of biometric sensors and outcomes. | Review of wearables used for monitoring cardiometabolic health, including sensors for BP, HR, ECG, glucose, and cholesterol. | The types of wearables used were smartwatches (45.3%), patches (34.0%), chest straps (22.6%), wristbands (13.2%), and others (9.4%). - HR (58.5%), glucose (28.3%), and ECG (26.4%) were the predominant indicators. No studies tracked BP or cholesterol. | Wearables offered features like physical activity, respiration, sleep, diet, and symptom monitoring. The impact of wearables on cardiometabolic indicators varied throughout the studies, indicating the need for further research. |
| 29 | DOI: 10.1109/ICHMS49158.2020.9209479 | Prediction of Personal Cardiovascular Risk using Machine Learning for Smartphone Application | Edmund Seto et al., 2020 | Experimental study | 5,992 adults | Used ML (generalized linear, stochastic gradient boosting, RF, and neural network models) applied to demographic, dietary, physical activity and mental health variables to predict cardiovascular risk factors (hypertension, body mass index, and TC level). | Developed an Application Programming Interface (API) for a mHealth smartphone app and web interface to demonstrate the use of ML models to provide personalised cardiovascular risk feedback. | Highest accuracy of 73% was found for predicting hypertension status using a RF model with demographic, diet, physical activity, behaviour, and mental state predictor variables. The API allows users to input predictor variables to compute a prediction of hypertension status. | ML-based personalised CVD risk prediction on smartphones is feasible and can enhance feedback; real-world benefit in prevention deserves further study. |
| 30 | DOI: 10.1056/NEJMra2301903 | Wearable Digital Health Technologies for Monitoring in Cardiovascular Medicine | Erica S. Spatz et al., 2024 | Systematic Literature Review | - | Reviewed several studies and focused on smartwatches, wearable ECGs, BP monitors and smartphone-applications and their potential in different CVD predictions or prevention. | Remote BP, rhythm and volume monitoring; application of consumer and implantable devices in routine and hospital care. | Wearables and remote monitors can detect AF (sensitivity 78–88%), BP, and volume status, allowing early detection and a more tailored management; clinical utility shown for arrhythmia detection, but no lipids were mentioned. | Digital health technologies offer new possibilities for continuous CVD management outside clinics; considerable challenges remain regarding accuracy, equity, and regulation. |
| 31 | DOI: 10.3390/jpm13121703 | Survey of Transfer Learning Approaches in the Machine Learning of Digital Health Sensing Data | Lina Chato et al., 2023 | Systematic Literature Review | - | Comprehensive review of transfer learning (TL) approaches in ML applied to digital health sensing data. | Discusses digital health technologies, including wearable devices, IoT/IoMT, and their role in healthcare. Explores various TL methods such as feature extraction, fine-tuning, domain adaptation, multitask learning, federated learning, and few-/single-/zero-shot learning. Highlights the challenges of using ML in digital health, including data fragmentation, privacy concerns, and bias. | TL is effective in addressing challenges like small datasets and fragmented data in digital health applications. TL methods improve the accuracy and efficiency of diagnoses and prognoses in healthcare. | Key limitations include the lack of standardized data formats, privacy issues, and the need for clinical validation of TL models. Future research should focus on developing new TL strategies to overcome these limitations and enhance digital health technologies. |
| 32 | - | Smart Healthcare Wearable Device for Early Disease Detection Using Machine Learning | Suraj S. Damre et al., 2022 | Experimental study | - | Developed a Smart Healthcare Wearable Device equipped with sensors to monitor vital signs such as HR, BP, oxygen saturation, and body temperature. Integrated advanced ML algorithms trained on large datasets to detect subtle deviations from baseline health patterns and identify early indicators of diseases. Implemented adaptive learning processes to tailor detection capabilities to individual users. | Designed an alert mechanism to notify wearers and healthcare providers of potential health concerns, enabling timely medical intervention. Provided personalized health recommendations for activity, sleep optimization, and dietary advice based on data-driven insights. | Demonstrated that ML algorithms can identify early deviations in multiple health parameters, potentially offering faster intervention for diseases. | The wearable device enables real-time monitoring of physiological parameters and early detection of health anomalies before symptoms manifest. The device promotes proactive wellness management and has the potential to improve treatment outcomes, reduce healthcare costs, and enhance quality of life. Collaboration among engineers, data scientists, healthcare practitioners, and ethicists is critical to address technical challenges, data security concerns, and ethical issues related to continuous health monitoring. |
| 33 | DOI: 10.1093/eurheartj/ehab874 | The year in cardiovascular medicine 2021 digital health and innovation | Panos E. Vardas et al., 2022 | Systematic Literature Review | - | Review on AI for ECG/arrhythmia/valvular disease, ML-based risk prediction, wearables for arrhythmia and physical activity tracking, remote patient monitoring, and fairness in digital health. | The article presents some of the most important developments in the field of digital medicine that have appeared over the previous 12 months and are related to cardiovascular medicine and concentrates on following main themes: artificial intelligence-enabled cardiovascular diagnostic tools, techniques and methodologies + big data and prognostic models for cardiovascular risk protection + wearable devices in cardiovascular risk assessment, CVD prevention, diagnosis and management. | AI-augmented ECGs and wearables perform at or above expert level in arrhythmia detection (AUC >0.9 reported), activity tracking enables new insights for AF risk, and ML risk models show improvement vs logistic regression in some settings. | Retinal photography is a non-invasive imaging modality that can provide information on the human vasculature and therefore CVD; DL algorithms can provide a coronary artery calcium score derived from retinal scan data. Digital health innovations, especially AI-enabled wearables/ECG and big data models, are transforming CVD prevention and management; robust clinical trials and attention to explainability and algorithmic equity remain crucial |
| 34 | DOI: 10.1016/j.jacc.2023.04.054 | Consumer Wearable Health and Fitness Technology in Cardiovascular Medicine | Bradley J. Petek et al., 2023 | Systematic Literature Review | - | Critical review of metrics (HR, HRV, SpO2, activity, sleep, cardiorespiratory fitness), accuracy, interpretation pitfalls, integration in clinical care. | Critically analyses common health metrics provided by consumer wearable devices (CWDs). Describes common pitfalls in CWD interpretation. Provides recommendations for the interpretation of abnormal results. Presents the utility of CWDs in exercise prescription. Examines health disparities and inequities in CWD use and development. Presents future directions for research and development. | Heterogeneous health metrics generated by CWDs make interpretation challenging. Physicians should become familiar with the measurement techniques, accuracy, clinical relevance, and potential pitfalls inherent in these devices as they continue to evolve. Most wearables enable reliable HR and activity monitoring. | Consumer wearable devices, when understood and used appropriately, offer new opportunities to improve cardiovascular health, but careful interpretation, patient guidance, and continual validation are essential. |
| 35 | DOI: 10.1016/j.sna.2023.114993 | A review on flexible wearables – Recent developments in non-invasive continuous health monitoring | Nikolay L. Kazanskiyet al., 2024 | Systematic Literature Review | - | Comprehensive literature research of non-invasive wearables with a technical focus. | Review of recent advances in flexible wearables for continuous non-invasive monitoring of HR, BP, body temperature, and blood glucose with a technical focus on sensor principles, materials, data types, integration and market trend. | Wearables provide accurate HR, activity and temperature measurements, some can detect non-invasively glucose. | By continuously collecting data on vital signs, physical activity, sleep patterns, and more, wearables empower users to gain valuable insights into their well-being, facilitating proactive health management. Despite existing challenges, the industry is well-positioned for ongoing expansion as it tackles these issues and pushes the frontiers of wearable technology. Challenges remain in integrating multimodal sensors, personalisation and user comfort |
| 36 | DOI: 10.1038/s42255-023-00778-y | Wearable and digital devices to monitor and treat metabolic diseases | Ayya Keshet et al., 2023 | Systematic Literature Review | - | Overview of wearable and digital devices monitoring parameters (CGM, ECG, PPG, physical activity, dietary/sleep logging apps) relevant for cardiometabolic diseases. | Integration of wearables and digital logging for real-time metabolic and CVD management. | Wearable and digital devices allow for frequent characterization of health-related outcomes, without the need for frequent clinic visits. Wearable devices provide unprecedented opportunities for monitoring and managing cardiometabolic diseases. | Discusses how data collected from such devices could help deepen our understanding of metabolic diseases, improve their diagnosis, identify early disease markers and contribute to individualisation of treatment and prevention plans. Integration of continuous-glucose-monitoring (CGM) data with data from insulin pumps and other wearable sensors could result in better glucose prediction and automatic insulin-modulation algorithms. |
| 37 | DOI: 10.33137/utmj.v100i2.41461 | The emerging role of wearables in cardiac care | Sophie Sigfstead et al., 2023 | Systematic Literature Review | - | Overview of available consumer wearable technology, its benefits and limitations, and its potential impact on cardiac care delivery and patient monitoring. | Consumer and clinical-grade wearable sensors (PPG, ECG), integration with AI and telemonitoring for detecting and monitoring cardiac arrhythmias and events. | Wearables can reliably detect arrhythmias (e.g., AF) in real-world settings, with population screening efficacy similar to traditional approaches. | Wearables have demonstrated value in diagnosing cardiac conditions, assisting with personalised disease management, improving health outcomes, and providing continual monitoring. Current limitations are related to issues such as inaccessibility and device inaccuracy. PPG technology could be a feasible approach for AF screening. Ultimately, the use of both PPG and ECG technologies is likely to provide consumers with the most comprehensive and useful information. |
